# Herpes simplex virus type 1 related acute retinal necrosis following an encephalitis illness: a case report

**DOI:** 10.1186/s12883-021-02082-2

**Published:** 2021-02-02

**Authors:** Pingting Zhong, Siwen Zang, Honghua Yu, Xiaohong Yang

**Affiliations:** 1 Department of Ophthalmology, Guangdong Eye Institute, Guangdong Provincial People’s Hospital, Guangdong Academy of Medical Sciences, Guangzhou, 510080 China; 2 Shantou University Medical College, Shantou, China

**Keywords:** Acute retinal necrosis, Encephalitis, Herpes simplex virus, Steroids, Antiviral treatment

## Abstract

**Background:**

Virus encephalitis is found to be a risk factor for acute retinal necrosis (ARN).

**Case presentation:**

We herein presented a case of a 20-year-old teenage boy who suffered from encephalitis of unknown etiology with early negative pathologic results, and was primarily treated with systemic administration of high-dose steroids without antiviral therapy. He later had sudden vision loss in his right eye. Intravitreal and intravenous antiviral treatments were immediately started due to suspected ARN. Herpes simplex virus (HSV)-1 was identified later in the vitreous humor of the patient. After the surgery of retinal detachment (RD), obvious improvements in vision were observed. However, the patient had recurrent RD and vision declination 5 weeks later.

**Conclusions:**

The case with suspected viral encephalitis should be treated with antiviral therapy regardless of early virologic results in order to avoid complications of a missed viral encephalitis diagnosis, especially if systemic steroid treatment is being considered.

## Background

Acute retinal necrosis (ARN) is a serious and potential blinding viral ocular infection, and it rapidly develops and progresses in immunocompetent people, causing uveitis with necrotizing retinitis [1]. Varicella-zoster virus (VZV) and herpes simplex virus (HSV) types 1 and 2 are the most common causative viruses of ARN [1]. It is assumed that reactivation amid immune dysfunction of the virus leads to ARN, along with central nervous system infection [2]. The association between viral encephalitis and ARN has been reported in one per 1.6–2.0 million people [3]. Therefore, additional attention with regard to ocular clinical manifestations is specially needed in patients with encephalitis after systemic treatment with steroids, as they could affect the body immunity and cause reactivation of the virus. Viral encephalitis should be aware of as it is a risk factor of ARN, and so antiviral treatment is recommended for suspected viral encephalitis.

## Case presentation

A 20-year-old teenage boy with abrupt fever, confusion, and epileptic seizures was referred to the Department of Neurology of the Guangzhou General Military Hospital. The patient was otherwise a healthy boy until he had an unusual health status prior to 5 days. Cerebral spinal fluid (CSF) analysis was performed on admission, and the result showed negative Pandy test with a predominance of lymphocytes. Besides, CSF test for *Mycobacterium tuberculosis*, herpes simplex virus (HSV), cytomegalovirus (CMV), and rubella virus revealed negative results. Two days later, antibody analysis of autoimmune encephalitis was also shown to be normal. Brain magnetic resonance imaging (MRI) showed hypersignal intensity in bilateral frontal as well as temporal lobe.

Next, the patient was diagnosed with encephalitis due to unidentified etiology, and then systemic administration of steroids was given without antiviral treatment. He was prescribed with intravenous methylprednisolone, 1 g/d for 3 consecutive days, followed by 0.5 g/d for 3 days, and then was maintained on 80 mg/d for 2 weeks. Two weeks after admission, the boy showed no sign of improvement. A second brain MRI showed much worsened manifestation of hypersignal in both bilateral frontal and temporal lobe, and a second CSF analysis showed negative results of the pathogen as shown in the first CSF analysis. He was therefore presumed to have “autoimmune encephalitis” by primary neurologists and was prescribed with gamma globulin 25 g/d for 5 days. Later, he had less fever and seizures, and improvements were observed in his oral expression. So, intravenous methylprednisolone administration was gradually reduced and replaced it by oral prednisone of 60 mg/d and then was discharged. One day after being discharged, he had sudden vision loss in his right eye, and then the boy was urgently referred to our hospital.

### Investigations

**Fig. 1:**
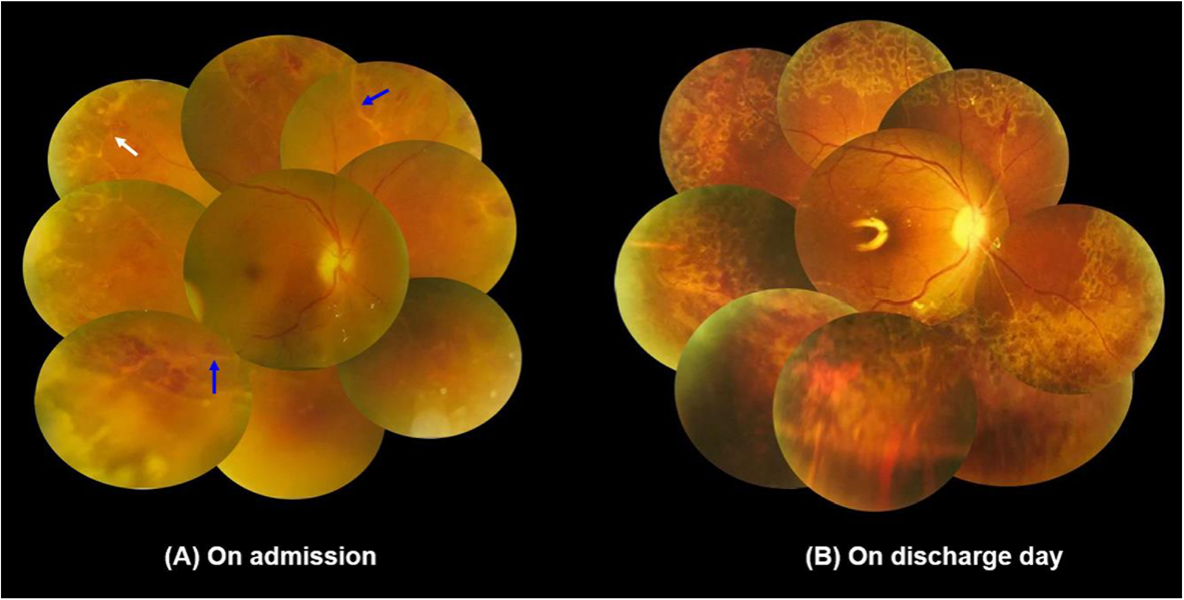
Fundus photographs of the right eye. **a** On admission. The blue arrows represent severe occlusive vasculitis, with macula involved in the peripheral retina, and white arrow represents several tiny holes on superior-nasal degeneration area. **b** On discharge day. Retinal detachment was repaired with retinal vasculitis and edema showed great improvement

After admitting in our hospital, the patient’s physical and neurological exams were found to be unremarkable. His best corrected visual acuity (BCVA) showed light perception with correct light location in the temporal region of the right eye (OD), and 20/20 with that of the left eye (OS). Ophthalmological examination of his right eye revealed positive Tyndall (+) and cell (++) in the anterior chamber, with obvious opacity (+++) in the vitreous chamber. The fundus of his right eye showed yellow-white lesions, with narrowing retinal vessels and white-sheath and peripheral hemorrhage. Also several tiny retinal holes that lead to retinal detachment (RD) were observed in the peripheral retina (Fig. 1a). No remarkable changes were observed in his left eye.

**Fig. 2:**
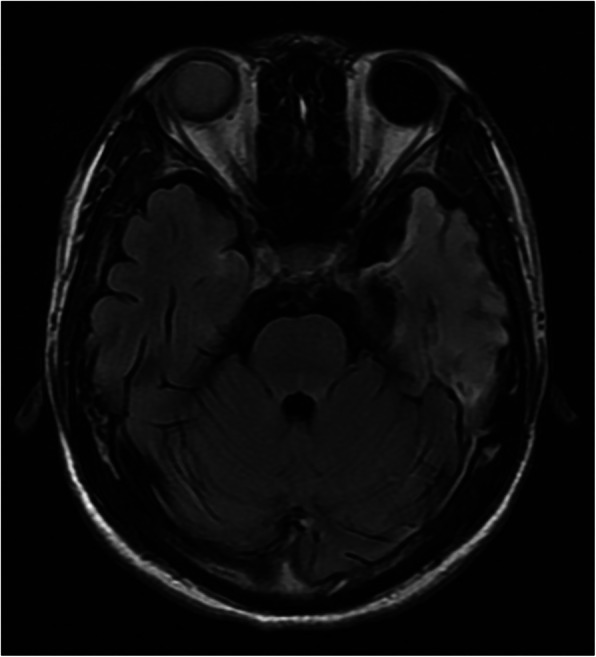
Hypersignal intensity of left temporal lobe in MRI with T2 flair

New brain MRI performed in our hospital suggested multiple abnormal signals in the brain parenchyma, which were in accordance with the manifestations of viral encephalitis (Fig. 2). So, viral-related retinal disorders were highly suspected in our case. Vitreous humor was obtained through vitreous chamber tapping to perform polymerase chain reaction (PCR) analysis. DNA of HSV-1 virus (9.0 × 10^6^/ml) was identified 5 days after intravitreal antiviral treatment, and the positive results of IgG and IgM antibodies in the blood serology also supported HSV-1 infection, thus confirming the diagnosis of ARN by HSV-1.

### Treatment

Intravitreal administration of ganciclovir (0.4 mg/ 0.1 ml) was immediately performed following vitreous chamber tapping at the time of admission to our hospital. Two days later, the boy was relieved from vitreous opacities (+). Antiviral treatment was therefore considered to be effective, and broad-spectrum antiviral medicine (ganciclovir 250 mg every 12-h) was started intravenously, and then replaced with intravenous acyclovir (500 mg every 8-h) after confirmation of HSV pathogen. As the patient also suffered from RD, his right eye was treated by pars plana vitrectomy (PPV), endolaser and silicone oil tamponade 3 days after admission. During the surgery, a second time intravitreal ganciclovir (0.4 mg/0.1 mg) was given.

### Outcomes and follow-up

**Fig. 3:**
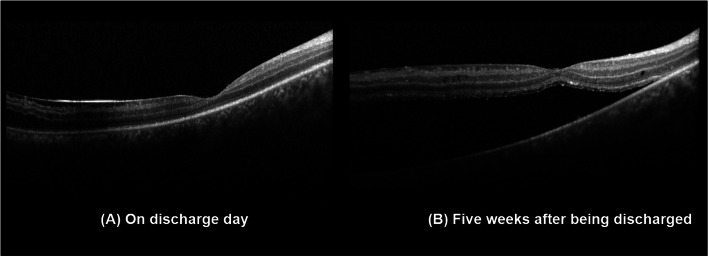
OCT images of the right eye. **a** On discharge day and **b** at 5 weeks after discharge

The patient received intravenous antiviral treatment for 2 weeks and was discharged with oral antiviral medicine (famciclovir 375 mg twice a day) as planned for 3 to 4 months. On the day of discharge, BCVA was 20/80 OD and retinal edema in his right eye has been greatly relieved (Fig. 1b). At 5 weeks of follow-up, recurrent vision declination occurred with 20/500 OD due to recurrent RD (Fig. 3). Therefore, silicon oil displacement and endolaser were performed to repair the retina, as well as intravitreal ganciclovir (0.4 mg/0.1 mg) was given for third time. The BCVA of his right eye was increased to 20/80 within 3 days after the surgery. Six months later, after removing the silicone oil, the BCVA was shown to be 20/200 OD with complicated cataract.

## Discussion and conclusion

Our patient due to encephalitis suffered from HSV related ARN after systemic administration of steroids. HSV-infected ARN could be a serious threat that leads to vision loss. Therefore, early awareness and timely antiviral treatment of suspected viral encephalitis are critical in such patients.

The possible reason for the cause of ARN in this patient might be due to viral encephalitis. HSV-1 virus encephalitis is usually characterized by altered mental health status, seizures, somnolence, increased cellularity with predominant lymphocytes in CSF, as well as hypersignal intensity in the MRI of temporal lobes [4], and all these clinical manifestations were observed in our patient. In our case, encephalitis was highly suspected to be caused by viral infection. However, lack of direct evidence of the virus in CSF impeded antiviral treatment. The patient later suffered from ARN due to HSV, suggesting that the virus might come from the brain. Due to the negative evidence in CSF, it could result in low positive predictive value [5] or procedural-related problems. To repeat CSF analysis is important in suspected viral encephalitis. ARN has been reported in cases with prior [6], simultaneous [7], or post [8] presence of herpetic simplex encephalitis or meningitis, and the interval between ARN and meningitis/encephalitis varied from 2 to 5 weeks [9]. A possible underlying mechanism has demonstrated bidirectional fast-axonal transport in neurons [10], and the viral genes play a critical role for antegrade and retrograde axonal transportation.

Immunocompromise after systemic administration of high-dose steroids could be another possible reason for the triggering of ARN in the current case. There are several possible explanations for steroids contributing to the occurrence of ARN. Firstly, high-dose steroids might affect body immunity, promote viral replication, and worsen necrotizing retinopathy [11]. The virus might reach the eye from the brain by a trans-axonal route. Secondly, the triggering event of systemic administration of high-dose steroids could reactivate HSV infection [12], and the latent HSV in several sites is connected to the eye, finally resulting in herpetic ocular disease that involves the cornea, iris, or even the retina [13]. When treating patients with encephalitis, for whom systemic administration of steroids is an inevitable regimen, neurologists should be aware that it might lead to immunocompromise, posing a serious threat in triggering ARN. In addition, according to prior studies on treatment of HSV1-encephalitis by combining with acyclovir, a study showed that treatment without corticosteroid was associated with poorer outcomes [14], while another study found no positive effects by adding dexamethasone to acyclovir [15]. Therefore, the use of corticosteroid therapy for viral encephalitis depends on the discretion of clinicians. As patients with encephalitis always present with confusion, which prevents them from timely and precise expression of their ocular discomforts, and so attention should be paid with regard to ocular clinical manifestations.

Intravitreal and intravenous antiviral treatment was then immediately started in this patient, and this is because of high suspicion of ARN according to ocular manifestations. So, diagnostic testing of vitreous humor before antiviral treatment has been done, and later corresponding adjustments were made. Topical and systemic antiviral treatment is an urgent need, as it is beneficial for the visual acuity and thus could decrease the risk of infection to the other eye [16]. As documented previously, there were up to 70% of untreated patients with bilateral ARN [17]. In our case, ARN was presented in a single eye, but it is assumed that the contralateral eye might also be affected if timely and precise antiviral treatment is not given. With a better understanding of antiviral treatment, the rate of bilateralization according to the recently reported studies on ARN has been found to be significantly decreased into 10–20% [18].

The challenges concerning diagnosis as well as prognosis were posed in this case. The CSF initially revealed negative results for viral encephalitis, and the diagnosis of ARN was later confirmed by PCR analysis with HSV-1 in the vitreous humor, and this is widely available to clinicians with good sensitivity and specificity [19]. According to a recent study, the correlation of quantitative DNA PCR and clinical prognosis in ARN has revealed that a number of copies superior to 5.0 × 10^6^/ml showed association with a higher probability of RD [20]. In our case, the quantitative DNA of HSV-1 was 9.0 × 10^6^/ml, suggesting a poor prognosis of vision in accordance with recurrent RD and vision declination during the follow-up period.

In the suspected case of viral encephalitis, antiviral therapy should be performed regardless of early PCR results to avoid complications of missed viral encephalitis, especially if systemic glucocorticoid therapy is being considered. Besides, special awareness and careful evaluation on neuro-ophthalmological assessment should be paid in any patients with a central nervous system disease. The clinical decision-making should be tailored to suit patients with ARN related to encephalitis, considering the extent and severity of the diseases and symptoms, as well as disease progression. Furthermore, consultation with a multidisciplinary team related to ophthalmology is highly recommended.

## Data Availability

All data generated or analyzed during this study are included in this article and are available from the corresponding author upon reasonable request.

## Abbreviations

ARN: Acute retinal necrosis
HSV: Herpes simplex virus
BCVA: Best corrected visual acuity
CSF: Cerebral spinal fluid
RD: Retinal detachment
PCR: Polymerase chain reaction
